# Fibroblastic galectin-1-fostered invasion and metastasis are mediated by TGF-β1-induced epithelial-mesenchymal transition in gastric cancer

**DOI:** 10.18632/aging.203295

**Published:** 2021-07-14

**Authors:** Xiaolan You, Jian Wu, Xiaojun Zhao, Xingyu Jiang, Wenxuan Tao, Zhiyi Chen, Chuanjiang Huang, Tingrui Zheng, Xianhe Shen

**Affiliations:** 1 Department of Gastrointestinal Surgery, Taizhou Clinical Medical School of Nanjing Medical University (Taizhou People’s Hospital), Taizhou 225300, Jiangsu, China; 2 Department of Clinical Speciality, Nanjing Medical University, Nanjing 210009, Jiangsu, China; 3 Department of Clinical Speciality, Southeast University, Nanjing 210009, Jiangsu, China

**Keywords:** galectin-1, gastric cancer microenvironment, cancer-associated fibroblasts, epithelial-mesenchymal transition, TGF-β1/Smad signaling pathway

## Abstract

Background The gastric cancer (GC) microenvironment has important effects on biological behaviors, such as tumor cell invasion and metastasis. However, the mechanism by which the GC microenvironment promotes GC cell invasion and metastasis is unknown. The present study aimed to clarify the effects and mechanism of galectin-1 (GAL-1, encoded by *LGALS1*) on GC invasion and metastasis in the GC microenvironment.

Methods The expression of GAL-1/ *LGALS1* was determined using western blotting, immunohistochemistry, and quantitative real-time reverse transcription PCR in GC tissues. Besides, methods including stable transfection, Matrigel invasion and migration assays, and wound-healing assays *in vitro*; and metastasis assays *in vivo*, were also conducted.

Results GAL-1 from cancer-associated fibroblasts (CAFs) induced the epithelial-mesenchymal transition (EMT) of GC cells though the transforming growth factor beta (TGF-β1)/ Sma- and mad-related protein (Smad) pathway, and affected the prognosis of patients with GC. The level of GAL-1 was high in CAFs, and treating MGC-803 and SGC -7901 cell line with the conditioned medium from CAFs promoted their invasion and metastasis abilities. Overexpression of *LGALS1* promoted the expression of TGF-β1 and induced EMT of GC cell lines. A TGF-β1 antagonist inhibited the invasion and migration of GC cells. *In vivo*, overexpression of *LGALS1* promoted GC growth and metastasis, and the TGF-β1 antagonist dramatically reversed these events.

Conclusions These findings suggested that high expression of GAL-1 in the GC microenvironment predicts a poor prognosis in patients with GC by promoting the migration and invasion of GC cells via EMT through the TGF-β1/Smad signaling pathway. The results might provide new therapeutic targets to treat GC.

## INTRODUCTION

In the digestive tract, one of the most malignant tumors is gastric cancer (GC), which has a high invasive ability. Worldwide, each year, there are more than 1 million new cases of GC, and among all malignant tumors, GC incidence and mortality rank fifth and third [1]. Thus, GC seriously threatens human health, particularly in Japan, South Korea, and China [2]. Although there has been considerable progress in the diagnosis and treatment of GC, the 5-year survival rate is still less than 30% in most countries owing to difficult early diagnosis and its high recurrence and metastasis rates [3]. Therefore, determining the molecular mechanisms of invasion and metastasis in GC, and seeking effective molecular markers for early diagnosis and prognosis are important to enhance GC diagnosis and treatment, and improve the survival rate of patients with GC.

For a long time, tumor biologists only focused on the biological characteristics of tumor cells, ignored the decisive role of the microenvironmental components of non-tumor cells in tumorigenesis and development, which stalled the development of anti-tumor therapies. Recently, researchers found that the tumor and its microenvironment form an integrated structure, and began to study of the effect of the microenvironment non-tumor cells on tumorigenesis and development. The tumor microenvironment (TME) is a complex and functional environment, which includes the extracellular matrix and multiple types of stromal cells, such as mesenchymal stem cells, macrophages, inflammatory cells, and fibroblasts. Fibroblasts can be activated into cancer-associated fibroblasts (CAFs) in the early stage of tumorigenesis, and become the most abundant cell type in the tumor stroma [4, 5]. Multiple proteins are highly expressed when CAFs are activated. At present, smooth muscle actin-α (SMA-α), which affects the motility of fibroblasts, is the most widely used marker of activated CAFs [6]. Recent studies have shown that CAFs are highly activated in GC tissues, and are closely associated with the malignant potential of GC, such as tumor size, tumor invasion, metastasis, metabolism, and remodeling [7]. Further studies confirmed that CAFs promote the tumorigenesis, development, invasion, metastasis, and other malignant potentials of GC cells via the secretion of various cytokines that act on GC cells [5, 8].

The lectin family member, galectin-1 (GAL-1, encoded by the *LGALS1* gene) is characterized by its affinity for glycans containing β galactosides [9]. In the TME, GAL-1 functions as a multivalent carbohydrate binding protein, cross-linking glycoproteins to mediate the activities of malignant cells [10]. For example, GAL-1 can cluster cell surface glycoproteins, form lattices and larger aggregates, and cross-link receptors thought to be involved in various mechanisms [10, 11]. GAL-1 activation occurs via by autocrine or paracrine sugar-dependent interactions with β-galactoside-containing glycoconjugates in the extracellular environment, and participates in tumor cell adhesion, migration, invasion, tumor-induced angiogenesis, and apoptosis via multiple interactions [12, 13]. Certain malignant tumors overexpress GAL-1, including GC [14]. Generally, in malignant tumor tissues with high expression of GAL-1 in the TME, interactions with sugar complexes regulate tumor progression. Specifically, in the TME, GAL-1 makes physical connections between the extracellular matrix and vascular endothelial cells, thus acting as a scaffold for vascular network formation and vascular growth, providing physical support for new vasculature [15–17]. However, how GAL-1 regulates GC invasion and metastasis in the GC microenvironment remains elusive.

GC invasion and metastasis is complex, involving many factors and steps. Epithelial-mesenchymal transition (EMT)-mediated downregulation of epithelial-associated markers, including E-cadherin, and upregulation of mesenchymal markers (e.g. Vimentin), has vital functions in GC metastasis and invasion [18]. EMT induces loss of polarity in epithelial cells, thereby decreasing their contacts with stromal and peripheral cells, and reducing intercellular interactions, which enhance cell migration and motility. Numerous signaling pathways are involved in EMT through cooperation and antagonism, such as transforming growth factor beta (TGF-β), Wnt/beta-catenin, and Ras-mitogen activated protein kinase (MAPKl) [19–21]. TGF-β is an important EMT-inducing factor during developmental processes and pathological states. TGF-β stimulation of certain cultured epithelial cell lines can induce EMT. TGF-β-induced EMT can occur via the classical Sma- and Mad-related protein (Smad) pathway or the non-Smad pathway. TGF-β activates Smad2 and Smad3, and combines with Smad4, in the classical Smad pathway. Then, Smad complexes will be transferred to the nucleus to mediate the inhibition or activation of target genes together with transcription factors. Meanwhile, Smad complexes also induce nuclear microRNA expression, which inhibits the signature protein expression of epithelial cells and promotes the expression of proteins that confer mesenchymal cell properties, which facilitates EMT [22]. TGF-β-mediated non-Smad signaling activates transcriptional regulation via the phosphatidylinositol-4,5-bisphosphate 3-kinase (PI3K)-protein kinase B (AKT)-mechanistic target of rapamycin (mTOR) signaling pathway [23]. Activation of AKT then inhibits ribonucleoprotein transcriptional regulation to trigger EMT [24].

Our previous study showed that GAL-1 promoted the invasion, metastasis, and vasculogenic mimicry of GC via EMT [25]. However, the TGF-β signaling pathway’s role in GAL-1-mediated promotion of GC EMT remains unclear. Clarifying the effects of GAL-1 on GC metastasis and invasion in GC microenvironment, and its molecular mechanism, will provide a new perspective and therapeutic targets to treat GC.

## RESULTS

### CAFs overexpresses GAL-1/ *LGALS1* and promotes lymph node metastasis in GC

**Figure 1:**
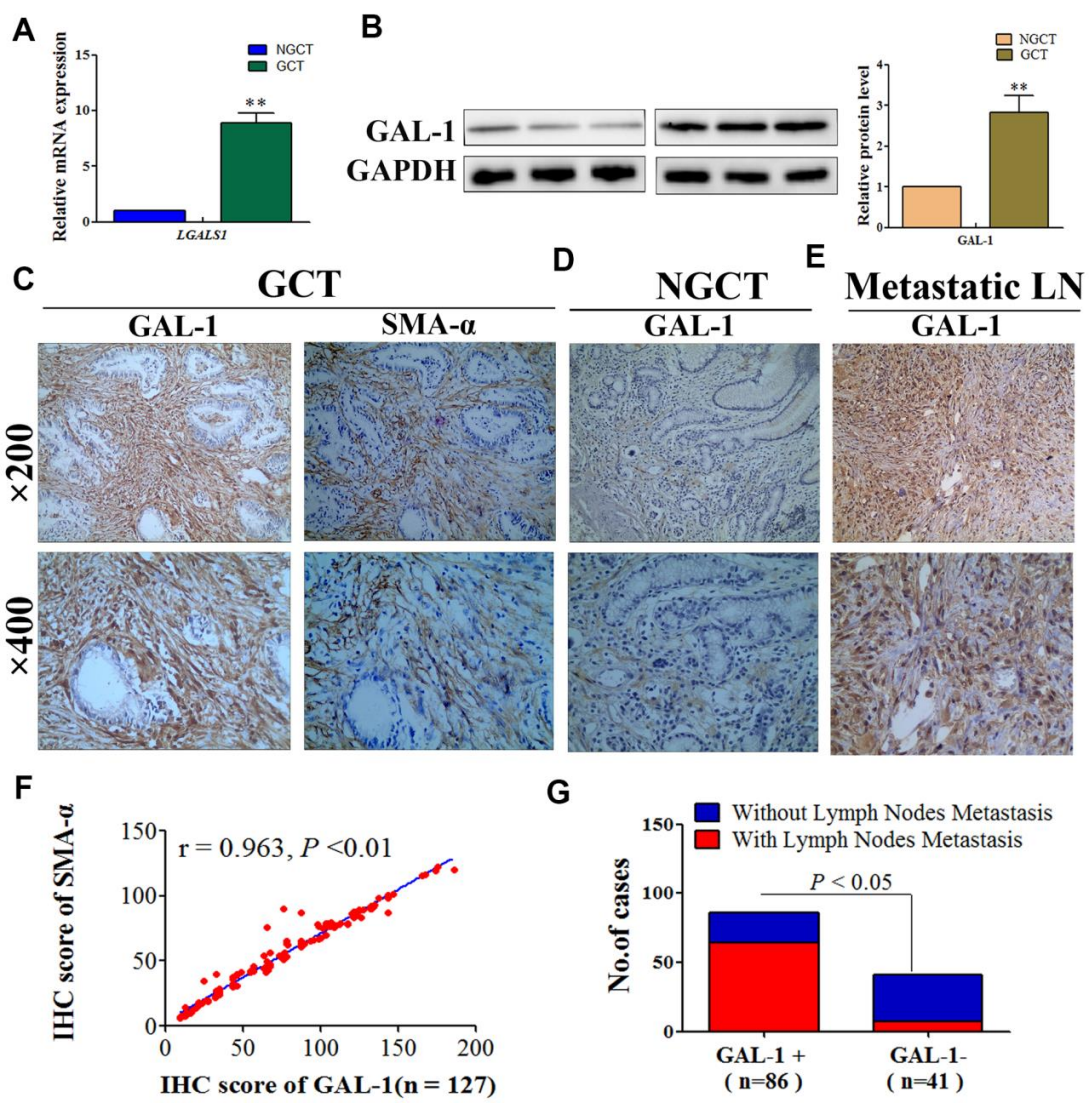
**GAL-1/*LGALS1* is overexpressed in CAFs and promotes lymph node metastasis in GC tissues.** (**A**) GCT exhibited significantly higher levels of the *LGALS1* mRNA than that in NGCT. (**B**) GAL-1 is overexpressed in GC tissues. (**C**–**E**) Representative images of IHC for GAL-1 and SMA-α protein levels in GCT, NGCT, and metastatic LN. (**F**) The IHC score of GAL-1 correlated positively with the IHC score of SMA-α in GC tissues (r = 0.963; *P* < 0.01). (**G**) The lymph node metastasis rate of the GAL-1-positive group was significantly higher than that in the GAL-1-negative group (*P* < 0.01).

To confirm that *LGALS1* is associated with the malignant behavior of GC, quantitative real-time reverse transcription PCR (qRT-PCR) was performed initially to assess *LGALS1* mRNA expression in 15 matched gastric cancer tissues (GCT) and non- gastric cancer tissues (NGCT). *LGALS1* mRNA expression was significantly different between NGCT and GCT: GCT exhibited significantly higher levels of *LGALS1* mRNA than that in NGCT (*P* < 0.01; Figure 1A). We then examined GAL-1 protein levels in 15 pairs of GCT and NGCT using western blotting (WB), the results of which were consistent with the qRT-PCR results, demonstrating higher levels of GAL-1 in GCT than in NGCT (*P* < 0.01; Figure 1B). Besides, the expression of GAL-1 was detected in GCT and in adjacent NGCT of 127 patients with GC using immunohistochemistry (IHC). Image Pro Plus (Media cybernetics, San Diego, CA, USA) was used to evaluated the digital images of IHC. GAL-1 median IHC scores in GCT and NGCT were 78.29 (9.51–186.24) and 31.09 (5.89–123.45), respectively. GAL-1 expression was significantly different between GCT and NGCT (Figure 1C, 1D; *P* < 0.01), and GAL-1 was mainly expressed by stroma tissue of GC (Figure 1C). To further confirm which cells produced GAL-1/ *LGALS1*, we used IHC to detect alpha smooth muscle actin (SMA-α) in GCT from 127 patients with GC. We found that GAL-1 was expressed in CAFs with a SMA-α protein positive phenotype (Figure 1C). Moreover, we found that GC cells expressed low or no GAL-1 (Figure 1C). Surprisingly, we found that the GAL-1 was highly expressed in GC cells from metastatic lymph node tissues when detected using IHC (Figure 1E). In GCT, SMA-α expression correlated positively with GAL-1 expression (r = 0.963, *P* < 0.01; Figure 1F), and residual analysis showed that GAL-1/SMA-α proteins fitted the regression model hypothesis (Supplementary Figure 1A). We classified the GAL-1 IHC scores as positive and negative using Receiver Operating Curve (ROC) statistics, and observed a significantly higher lymph node metastasis rate in the GAL-1 positive group (64/86) compared with that in the GAL-1 negative group (7/41) (*P* < 0.01; Figure 1G).

### CAFs express GAL-1/*LGALS1* and promote GC cell line metastasis and invasion abilities *in vitro*

**Figure 2:**
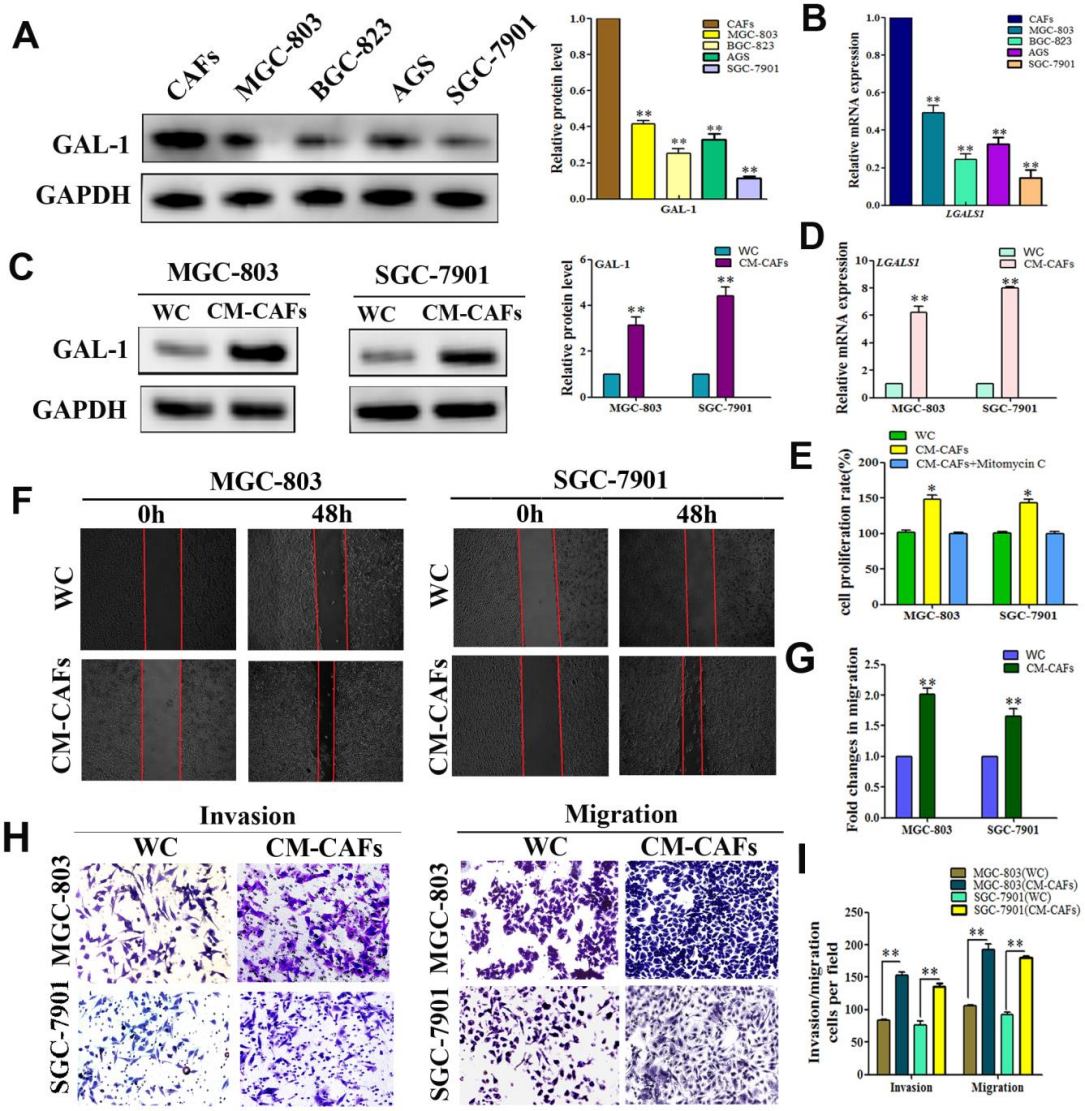
**CAFs expression of GAL-1/ *LGALS1* promotes the invasion and metastasis ability of GC cell lines *in vitro*.** (**A**, **B**) GAL-1 protein and *LGALS1* mRNA levels in CAFs and GC cell lines, GAL-1/*LGALS1* levels were high in CAFs. (**C**, **D**) Treatment of MGC-803 and SGC-7901 cells with CM-CAFs significantly increased GAL-1 protein levels and *LGALS1* mRNA expression. (**E**) CM-CAFs increased the proliferation of MGC-803 and SGC-7901 cells, and the proliferation effect was abolished when the medium contained 10 μg/mL mitomycin C. (**F**, **G**) MGC-803 and SGC-7901 cells treated with CM-CAFs exhibited a significantly enhanced migration capacity compared with the wild-type control (P < 0.01). Magnification: ×40. (**H**, **I**) Transwell assay showing that MGC-803 and SGC-7901 cells treated with CM-CAFs had increased cell invasion and migration abilities (*P* < 0.01). Magnification: ×200.

To further clarify the origin of GAL-1/*LGALS1*, we cultured CAFs from human GC tissue, and the GC cell lines (SGC-7901, AGS, BGC-823, and MGC-803) *in vitro* and investigated the GAL-1 levels in all cells using WB, which confirmed strong GAL-1 expression in CAFs, but lower levels in GC cells (Figure 2A). Meanwhile, the results of qRT-PCR analysis of *LGALS1* mRNA expression in all the cell lines were consistent with the WB results (Figure 2B). MGC-803 cells and SGC-7901 were then treated with conditioned medium (CM) from CAFs (CM-CAFs) for 72 h, and then GAL-1 and *LGALS1* mRNA levels were monitored using WB and qRT-PCR. Compared with untreated MGC-803 and SGC-7901 cells, CM-CAFs treatment of SGC-7901 and MGC-803 cells resulted in significant increases in the GAL-1 protein and *LGALS1* mRNA levels (Figure 2C, 2D). SGC-7901 and MGC-803 cell proliferation increased after CM-CAFs treatment for 48 h, and the proliferation effect was abolished when the medium contained 10 μg/mL mitomycin C (Figure 2E). CM-CAFs treatment of SGC-7901 and MGC-803 cells resulted in significantly increased migration abilities compared with those of the wild-type (untreated) control (Figure 2F); the fold changes in migration are shown in Figure 2G (*P* < 0.01). Transwell assays showed that MGC-803 and SGC-7901 cells treated with CM-CAFs had increased cell invasion and migration abilities (*P* < 0.01, Figure 2H, 2I).

### GAL-1/*LGALS1* promotes EMT in GC, and EMT promotes lymph node metastasis of GC

**Figure 3:**
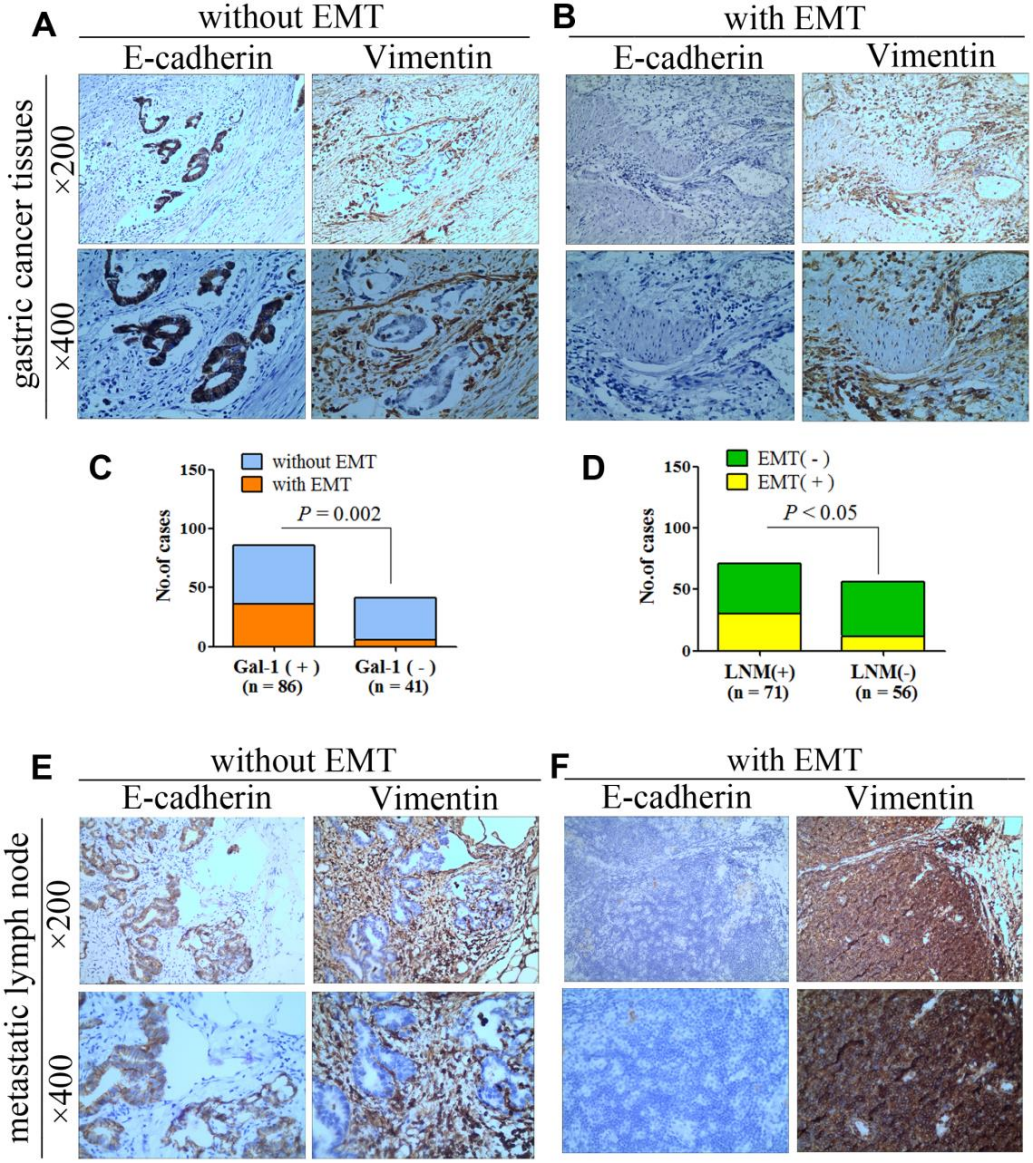
**GAL-1/*LGALS1* promotes EMT in GC, and EMT promotes lymph node metastasis of GC.** (**A**) Representative images of GCT without EMT. (**B**) Representative images of GCT with EMT. (**C**) GAL-1/*LGALS1* significantly promoted EMT in GC (*P* < 0.05). (**D**) GC with EMT was more prone to lymph node metastasis (*P* < 0.05). (**E**, **F**) Representative images of GC cells with or without EMT in metastatic lymph nodes.

To determine whether GAL-1/*LGALS1* promotes the migration and invasion of GC cells in an EMT-dependent manner, IHC of GC tissue was used to examine the EMT-related biomarkers Vimentin and E-Cadherin. In 85 cases, GC cells stained positive for E-cadherin and stromal cells stained positive for Vimentin, indicating that EMT had not occurred (Figure 3A). In 42 cases, GC and stromal cells stained positive for Vimentin, and E-cadherin levels were reduced, indicating that EMT had occurred (Figure 3B). The EMT rate in the GAL-1 positive group was 41.86% (36/86), while that in the GAL-1 negative group was 14.63% (6/41), suggesting that GAL-1 promotes EMT significantly in GC (*P* < 0.05, Figure 3C). In addition, we found that GC cases with EMT were more prone to lymph node metastasis: Among 71 patients with GC had lymph node metastasis, 30 with EMT (42.25%, 30/71), while there were 12 cases with EMT among 56 cases of GC hadn’t lymph node metastasis (21.43%, 12/56) (*P* < 0.05, Figure 3D). GC cells with or without EMT in metastatic lymph nodes are shown in Figure 3E, 3F.

### GAL-1/*LGALS1* activates TGF-β/Smad signaling pathways in GC tissues

**Figure 4:**
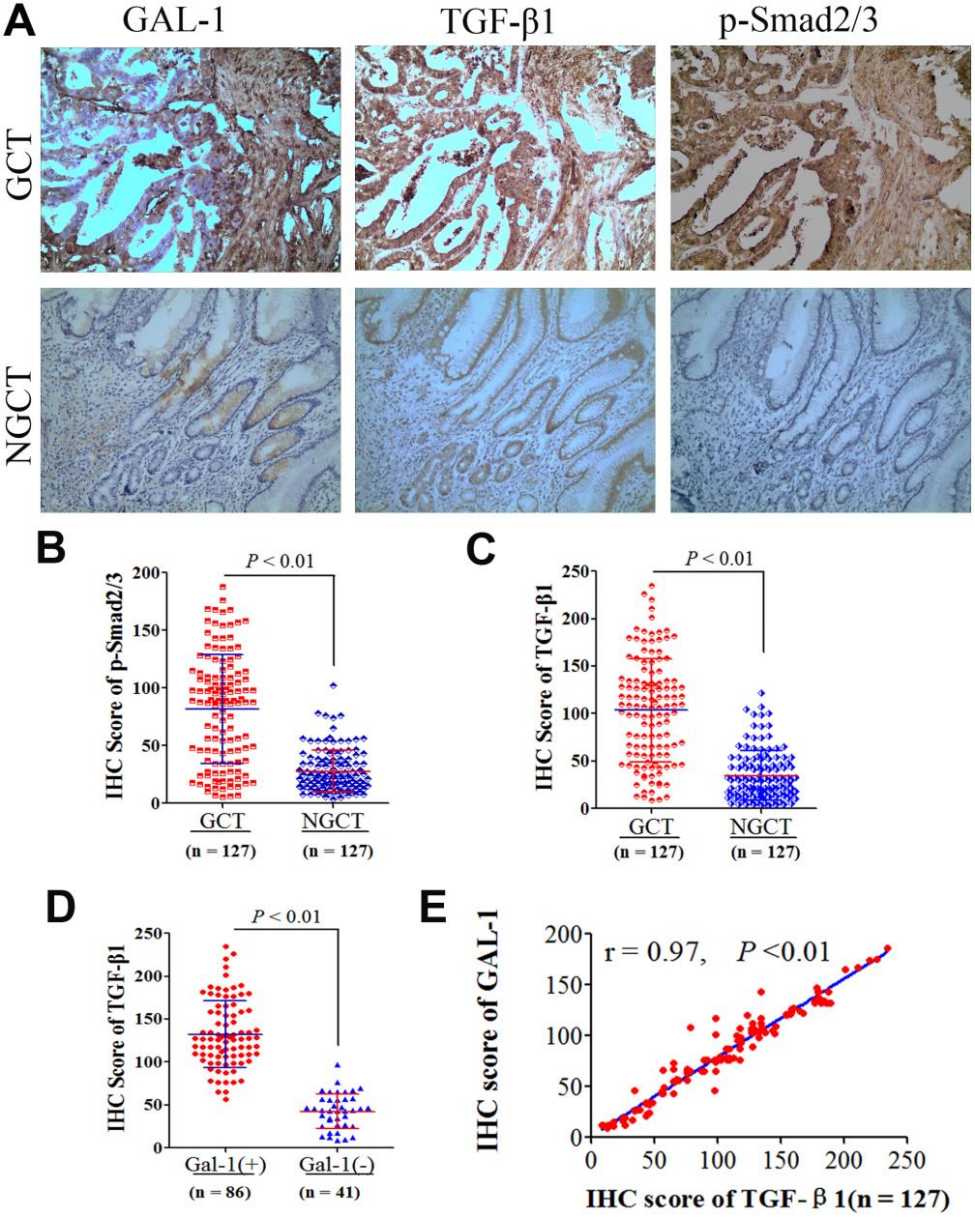
**GAL-1/*LGALS1* activates TGF-β/Smad signaling pathways in GC tissues.** (**A**) Representative images of IHC for GAL-1, TGF-β1, and p-Smad2/3 protein levels in GCT and NGCT. Magnification: ×400. (**B**, **C**) Significant differences in TGF-β1 and p-Smad2/3 levels were observed between GCT and NGCT (all *P* < 0.01). (**D**) The TGF-β1 IHC scores in GAL-1-positive GCT were significantly higher than those in GAL-1-negative GCT (*P* < 0.01). (**E**) The TGF-β1 IHC scores correlate positively with the GAL-1 IHC scores in GC tissues (r = 0.97; *P* < 0.01).

To investigate how GAL-1/*LGALS1* promotes EMT in GC mechanistically, we examined the protein levels of GAL-1, TGF-β1, and phosphorylated (p)-Smad2/3 in GCT and NGCT using IHC. Representative IHC images of IHC for p-Smad2/3, TGF-β1, and GAL-1, protein levels in GCT and NGCT are shown in Figure 4A. GCT and NGCT showed significant differences in p-Smad2/3 and TGF-β1 levels (*P* < 0.01; Figure 4B, 4C). For TGF-β1, the median IHC score for was 108.26 (8.741–234.56) in GC tissues, 127.22 (56.67–234.56) in GAL-1-positive GC tissues, and 43.47 (8.741–97.12) in GAL-1-negative GCT. In GAL-1-positive GCT, the IHC scores for TGF-β1 were significantly higher than those in GAL-1-negative GCT (*P* <0.01; Figure 4D). In GC tissues, the TGF-β1 IHC scores correlated positively with those of GAL-1 (r = 0.97; *P* < 0.01; Figure 4E). Residual analysis demonstrated that GAL-1/TGF-β1 proteins levels fitted the regression model hypothesis (Supplementary Figure 1B).

### GAL-1/*LGALS1* induces EMT via TGF-β/Smad pathways *in vitro*

**Figure 5:**
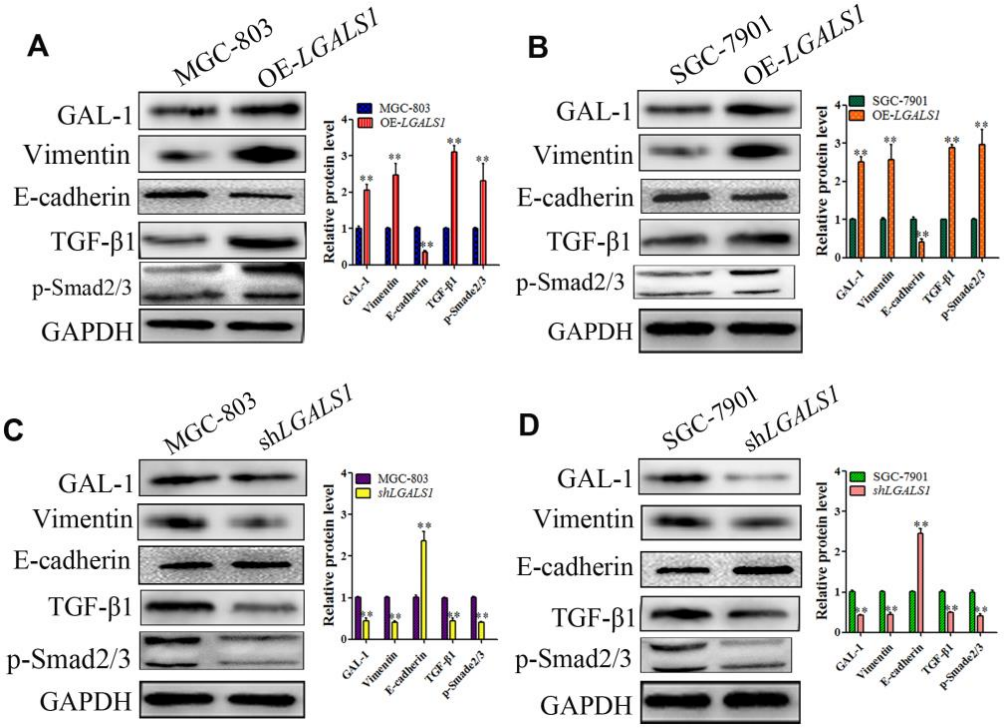
**GAL-1/ *LGALS1* induces EMT through TGF-β/Smad signaling pathways *in vitro*.** (**A**, **B**) WB showing that OE-*LGALS1* efficiently increased the expression of TGF-β1 and p-Smad2/3 and induced EMT in MGC-803 and SGC-7901 cells compared with the wild-type cells (all *P* < 0.01). (**C**, **D**) Silencing *LGALS1* in MGC-803 and SGA-7901 cells efficiently decreased the levels of TGF-β1 and p-Smad2/3 and inhibited EMT (all *P* < 0.01).

To identify if GAL-1 activates Smad/TGF-β signaling to induce EMT in GC, SGC-7901 and MGC-803 cells were transfected using LV- *LGALS1*-OE or LV-LGALS1-RNAi to overexpress or silence *LGALS1*, respectively, as described previously [16]. Western blotting showed increased levels of Vimentin and decreased levels of E-cadherin in MGC-803 cells overexpressing *LGALS1* (OE-*LGALS1*) compared with those in non-transfected MGC-803 cells (wild-type), and the levels of TGF-β1 and p-Smad2/3 also increased in MGC-803 OE-*LGALS1* cells (all *P* < 0.01, Figure 5A). The results in SGC-7901 cells were consistent with those in MGC-803 cells (all *P* < 0.01, Figure 5B). In MGC-803 cells silenced for *LGALS1*, E-cadherin levels increased, and Vimentin, TGF-β1, and p-Smad2/3 levels decreased (all *P* < 0.01, Figure 5C). SGC-7901 cells showed similar results (all *P* < 0.01, Figure 5D).

### In GC cells, GAL-1/*LGALS1* promotes invasion and migration *in vitro* via the TGF-β/Smad pathways

**Figure 6:**
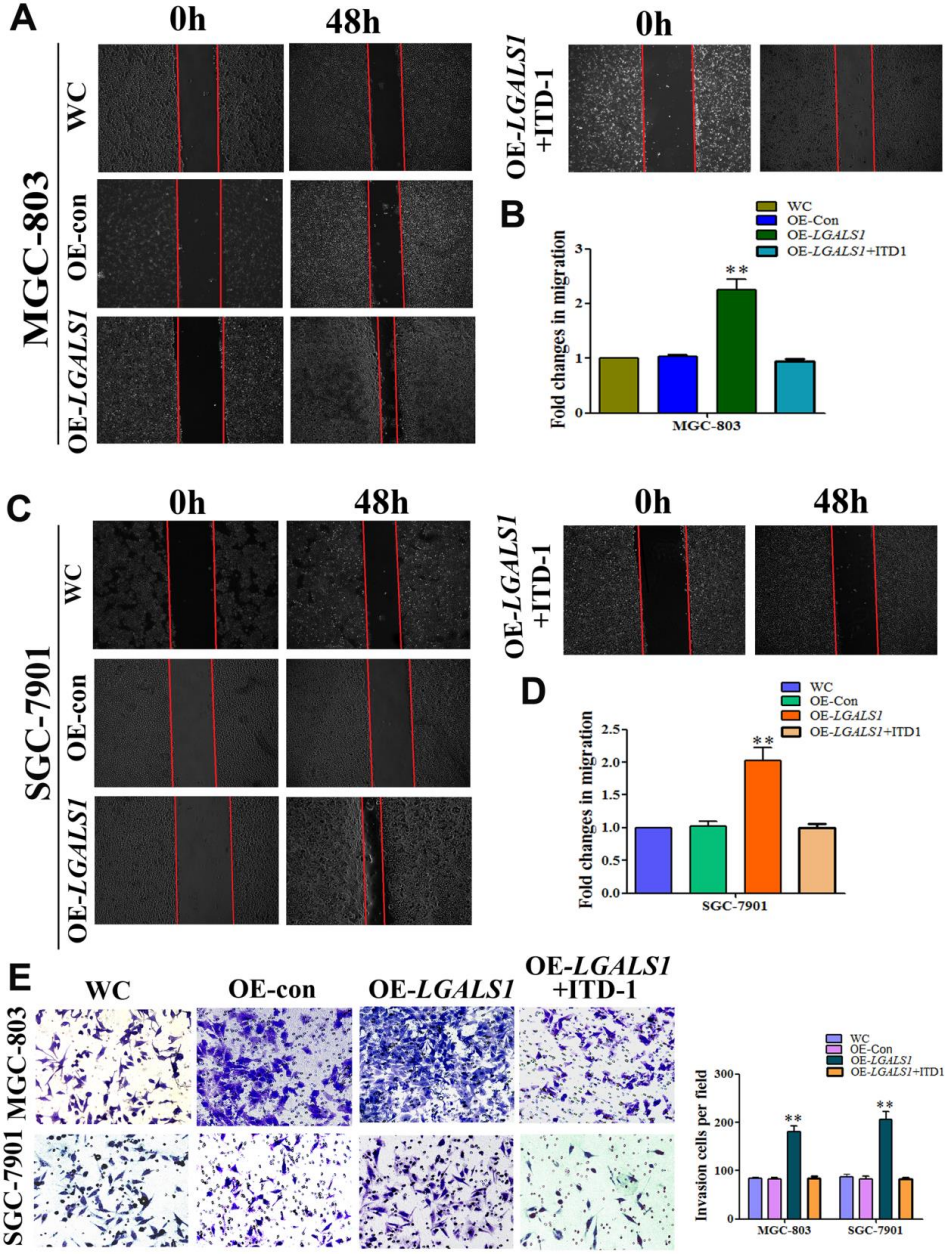
**GAL-1/ *LGALS1* promotes the migration and invasion of GC cells *in vitro* through TGF-β/Smad signaling pathways.** (**A**–**D**) OE-*LGALS1* significantly enhanced the migration capacity of MGC-803 and SGC-7901 cells compared with WC and OE-con. The migration capacity was abolished when the medium contained 10 μM ITD1 (*P* < 0.01). Magnification: ×40. (**E**) Transwell assay showing that MGC-803 and SGC-7901 cells increased their invasive ability after transfection with LV-*LGALS1*-OE, and 10μM ITD1 abolished this increase in invasive ability (n = 3). Magnification: ×200.

To investigate whether GAL-1/*LGALS1* promotes GC invasion and migration by activating TGF-β/Smad signaling, ITD1, a specific antagonist of TGF-β/Smad signaling was employed to investigate the relationship between the induction of GC cell migration and invasion by GAL-1 and TGF-β/Smad pathway activation. 3-(4,5-dimethylthiazol-2-yl)-2,5-diphenyltetrazolium bromide (MTT) assays suggested that neither ITD1 at 10 μM nor the empty cassette of the viral vector system affected the cell number at 48 h. OE-*LGALS1* increased the number of cells; however, the proliferation effect was abolished using 10 μg/mL mitomycin C. Compared with the negative control-transfected MGC-803 cells (OE-con) and wild-type control (WC) cells, OE-*LGALS1* MGC-803 cells showed significantly increased migration (*P* < 0.01, Figure 6A). However, this migration capacity was abolished when the medium contained 10 μM ITD1(*P* < 0.01, Figure 6A). The fold changes in migration are shown in Figure 6B.

To confirm GAL-1-mediated promotion of migration *in vitro*, these experiments were repeated in SGC-7901 cells. The migration of OE-*LGALS1* SGC-7901 cells was enhanced compared with that of the WC and OE-con SGC-7901 groups, and 10 μM ITD1 abolished this migration capacity (Figure 6C, 6D). As shown in Figure 6E, Transwell assays demonstrated that LV-LGALS1-OE-transfected SGC-7901 and MGC-803 cells had increased invasiveness, and the addition of 10 μM ITD1 abolished this capacity for invasion.

### GAL-1/*LGALS1* promotes GC cell metastasis via TGF-β/Smad signaling *in vivo*

**Figure 7:**
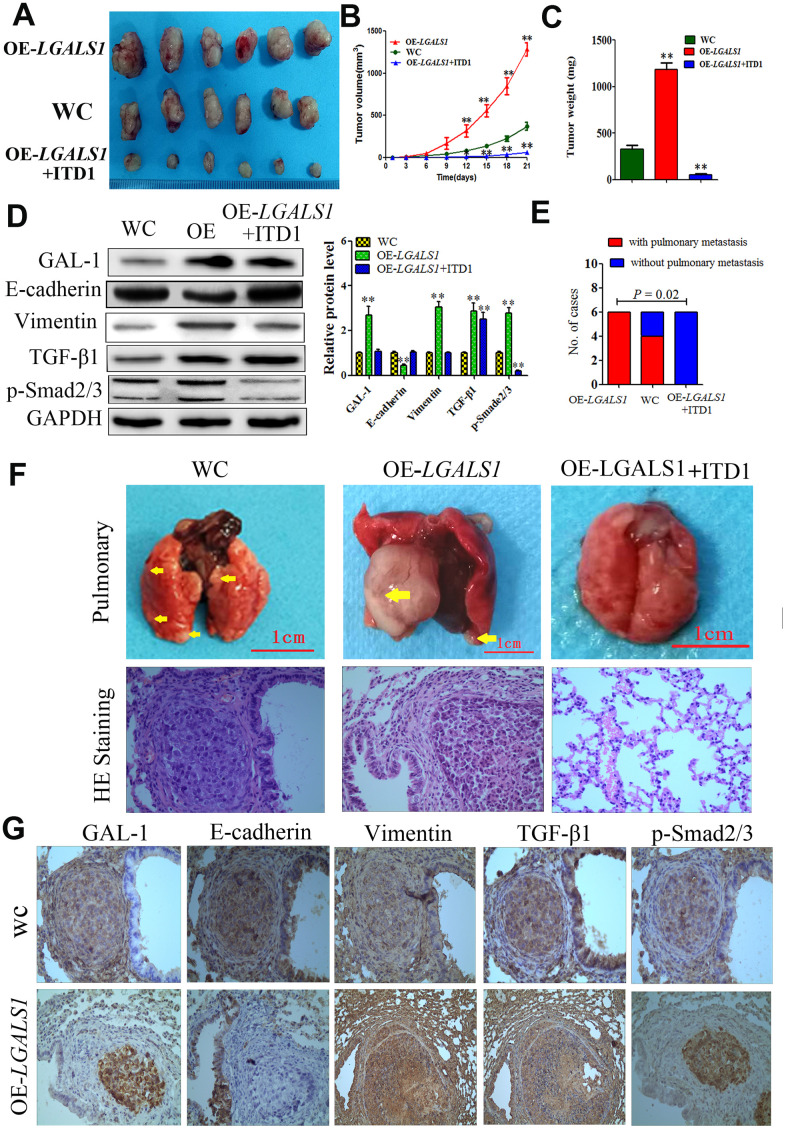
**GAL-1/ *LGALS1* promotes GC cell metastasis *in vivo* through the TGF-β/Smad signaling pathway.** (**A**) OE-*LGALS1* induced MGC-803 to form subcutaneous xenograft tumors with larger volumes (**B**) and weighs (**C**) (expressed as the mean ± SE). * *P* <0.05, ** *P*< 0.01, n = 6. (**D**) OE-*LGALS1* increased the levels of TGF-β1 and p-Smad2/3, and induced EMT in the subcutaneous xenograft tumor, ITD1 could inhibit this effect. Metastases were frequent in the (**E**). (**F**) Representative images of metastasis (yellow arrows) in the lungs at 50 days after inoculation, and representative images of H&E staining. Original magnification: ×400. (**G**) Immunostaining showing GAL-1, E-cadherin, vimentin, TGF-β1 and p-Smad2/3 levels in pulmonary metastases. Magnification: ×400.

*In vitro* experiments and clinical analysis showed that GAL-1/*LGALS1* promotes EMT-mediated enhancement of GC metastasis, invasion, and migration via TGF-β/Smad signaling. We established lung metastasis and subcutaneous GC implantation models in athymic mice (n = 6 per group) to further determine whether GAL-1/*LGALS1* promotes GC growth and metastasis through EMT induced by activating TGF-β/Smad signaling. At 21 days after subcutaneous implantation of GC cells, in the *LGALS1* overexpression group (OE-*LGALS1*), the tumors were larger and heavier compared with those in the WC group. Significantly smaller and lighter tumors were formed by OE-*LGALS1* MGC-803 cells treated with ITD1 compared with those formed in the WC group (Figure 7A–7C; *P* < 0.01). After day 12, the tumors in the OE-*LGALS1* group had a significantly larger volume compared with those in the WC group, and the tumor volumes in the OE-*LGALS1* MGC-803 cells treated with ITD1 were significantly lower than those in the WC group (*P* < 0.01 and *P* < 0.05; Figure 7B). According to the western blotting results, Vimentin levels in the OE-*LGALS1* group were increased, and E-cadherin levels were decreased, which suggested that *LGALS1* overexpression promoted EMT. Compared with those in the WC group, TGF-β1 and p-Smad2/3 levels were elevated in the OE-*LGALS1* group (*P* < 0.01, Figure 7D). However, ITD1 treatment reduced the levels of p-Smad2/3 significantly (*P* < 0.01, Figure 7D), and inhibited EMT in the implanted GC cells (Figure 7D).

In all the mice from the OE-*LGALS1* group, larger pulmonary metastases were observed after fifty days in the lung metastasis models. Four cases of pulmonary metastasis were found in the WC group, while no pulmonary metastases were observed in the ITD1-treated OE-*LGALS1* group (*P* = 0.002; Figure 7E). Hematoxylin and eosin (H&E) staining was used to identify pulmonary metastases in all groups (Figure 7F). IHC staining of TGF-β/Smad markers in pulmonary metastases, showed significantly increased GAL-1, Vimentin, TGF-β1, and p-Smad 2/3 levels and decreased E-cadherin levels in the pulmonary metastatic tissues from the OE-*LGALS1* group (Figure 7G). Our results suggested an important role of GAL-1 in GC metastasis and EMT, and the TGF-β/Smad signaling pathway potentially contributes to this process.

## DISCUSSION

Malignant tumor growth and metastasis is a complex and multi-step process. Recent studies show that a variety of growth factors and cytokines released by tumor stromal cells create a microenvironment suitable for tumor growth, which promotes tumor proliferation, growth, invasion, and escape from immune surveillance, leading to distant metastasis [26]. GAL-1, a multivalent carbohydrate-binding protein, regulates malignant tumor cell activity by cross-linking glycoproteins in the TME [27]. GAL-1 is synthesized on cytoplasmic ribosomes with a prototypical acetylated N-terminus, but no signal peptide [28], then GAL-1 is transported from the nucleus to the medial side of the cell membrane and secreted into the extracellular TME [28, 29]. In the extracellular environment, GAL-1 has high affinity with β-galactosides [28], and regulates cancer cell homotypic aggregation by interacting with sugar complexes on the cell surface [9]. Furthermore, GAL-1 can mediate the tumor cell adhesion to the extracellular matrix [13]. Glycoproteins in the baseman membrane, including laminin and fibronectin, provide GAL-1 binding sites that crosslink cells with the extracellular matrix; therefore, GAL-1 regulates the adhesion of cancer cells during metastasis through glycoproteins [13]. Conversely, GAL-1 inhibits the adhesion between the extracellular matrix and tumor cells by competitive binding with cell surface sugar complexes or matrix glycoproteins [30]. This suggests that GAL-1 has a role in the regulation of tumor cell isolation, a process by which tumor cells become detached from the primary site and then migrate to secondary sites [30]. Thus, GAL-1 regulates the adhesion between the extracellular matrix and tumor cells, regulates the binding of glycoproteins to the extracellular matrix, and enhances the activities of proteolytic enzymes, thereby promoting the metastasis of tumor cells [13].

GAL-1 has been found in multiple tumor cells, including melanoma, lung cancer [31], pancreatic cancer [32], bladder cancer [33], thyroid cancer [34], cervical cancer [35], and colorectal cancer [36]. The results of the present study demonstrated that the GAL-1 protein and *LGALS1* mRNA levels in GC tissue were significantly higher than those in NGCT, suggesting that GAL-1/*LGALS1* is associated with the malignant biological behavior of GC. We also observed that the GAL-1 in GC tissue is secreted by SMA-α-positive CAFs in the tumor microenvironment. WB and qRT-PCR also confirmed high GAL-1 expression in CAFs. Intriguingly, low or no GAL-1 expression was observed in GCT, but GC cells in metastatic lymph nodes showed high expression of GAL-1. Moreover, treatment of GC cells with CAFs cell conditioned medium increased GAL-1 expression in MGC803 and SGC-7901 cell lines, and promoted the invasion and metastasis of GC cell lines significantly. This indicated that GAL-1/*LGALS1* promotes GC cells to acquire a metastatic phenotype and enhances GC cell metastasis. However, how GAL-1/ *LGALS1* regulates the invasion and metastasis of GC in the TME remains mostly unknown.

For the invasion and metastasis of malignant tumors, EMT is a vital step [21], prompting us to hypothesize that GAL-1/*LGALS1* via EMT promotes GC invasion and metastasis. This hypothesis was tested by examining the EMT-related biomarkers Vimentin and E-Cadherin in GC and metastatic lymph nodes with IHC, which showed that the level of GAL-1 was related to EMT in GCT. In addition, we found that GC with EMT was more susceptible to lymph node metastasis.

The TGF-β family plays a vital role in EMT regulation [24]. The results of our IHC test also showed significantly higher levels of p-Smad 2/3 and TGF-β1 in GC tissue than in gastric mucosal tissue, and the level of TGF-β1 in GCT with GAL-1 positive expression was significantly higher than that in GAL-1 negative expression tissues. Moreover, the expression of GAL-1 in GCT correlated positively with TGF-β1 expression. Thus, in the GC microenvironment, GAL-1 potentially activates the TGF-β/Smad signaling pathway.

TGF-β1 is the typical member of a large family of functionally and structurally related proteins, including TGF-β2 and TGF-β3, bone morphogenetic proteins (BMPs), Mullerian-inhibiting substance (MIS), and growth and differentiation factors (GDF) [37]. In the 1980s, the growth of rat kidney cells was induced in soft agar by TGF-β1, identifying it as a secreted factor. TGF-β1 transmits signals from cell surface receptors to the nucleus, in which the Smads family of proteins play key roles. The Smads family are divided into inhibitory I-Smads (Smad -6 and -7), he common (Co-) Smads (Smad4), and receptor-regulated (R-) Smads (R-SMAD1, -2, -3, -5 and -8). R-Smads interact with activated TGF-β type I receptor kinases, and become phosphorylated. Activated R-SMADs and Co-Smads form heteromeric complexes, while I-Smads antagonize canonical Smad signaling [38]. In canonical Smad signaling, cell surface TGF-β type I and type II receptors mediate the cellular effects of TGF-β1. The binding of TGF-β1 to type II receptors results in the recruitment and phosphorylation of type I receptors. The type I receptors then rephosphorylate R-Smad proteins, which bind to Co-Smad. The complex of R-Smad/Co-Smad aggregates in the nucleus, where it functions as a transcription factor in cooperation with other transcription regulators, thus modulating the expression of its target genes. Therefore, in TGF-β family receptor signaling, Smad2/3 function as intracellular transcriptional effectors.

In the present study, IHC examination of GC tissue showed that the GAL-1 levels in the GC microenvironment correlated positively with TGF-β1 in GCT, and GCT with high GAL-1 expression had significantly higher levels of p-Smad2/3. P-Smad2/3 and TGF-β1 levels increased in GC cell lines overexpressing *LGALS1*, which underwent EMT and showed enhanced invasion and migration abilities. The overexpression of *LGALS1* promoted subcutaneous tumor growth and lung metastasis in nude mice. This subcutaneous growth and lung metastasis was inhibited in nude mice when the ITD1, a specific inhibitor of the TGF-β signaling pathway, was used to treat the cell lines. This further confirmed that GC invasion and metastasis is promoted by GAL-1/*LGALS1* via TGF-β/Smad signaling.

In human cells, TGF-β receptors are expressed widely, mediating a variety of biological processes, including tissue repair, immune surveillance, organogenesis, and embryonic development. Alterations in TGF-β signaling lead to many diseases, such as cancer. In the early stage of malignant tumor development, TGF-β signaling inhibits tumorigenesis by inducing apoptosis of premalignant cells. Meanwhile, cancer cells that acquire oncogenic mutations become resistant to apoptosis induced by TGF-β. TGF-β can induce tumor cells to undergo EMT, leading to metastasis and chemotherapy resistance [39].

Cancer cells escape from the primary site mainly via EMT-induced alterations to tumor cells and the changes in the TME [40]. In the TME, tumor necrosis factor-α, interleukin-6, chemokine 4/12, and TGF-β enhance EMT. EMT causes more epithelial growth factors to be secreted by tumor cells, leading to an acidic and hypoxic microenvironment with a high interstitial fluid pressure, which activates CAFs [41]. The present study showed that in GC, activated CAFs secrete GAL-1, which activates TGF-β/Smad signaling, thus promoting EMT. This represents a positive feedback loop that ultimately promotes GC invasion and metastasis.

Analysis of clinical specimens and the *in vitro* and *in vivo* experiments all confirmed that GAL-1 promotes GC invasion and metastasis via TGF-β/Smad signaling. However, the mechanism by which GAL-1/*LGALS1* activates TGF-β/Smad signaling in the GC microenvironment remains elusive. Further experiments are required to reveal its specific molecular mechanism to provide new targets for targeted therapy of GC.

## CONCLUSIONS

The results of the present study together suggest that fibroblastic GAL-1 promotes GC cells to acquire a metastatic phenotype, leading to lymph node metastasis in the GC microenvironment. In GC, components of the TGF-β/Smad signaling pathway are activated by GAL-1, resulting in EMT, which controls the initiation of GC invasion and metastasis. These results evidenced that the TME and the GAL-1/TGF-β/Smad pathway plays important roles in invasion and metastasis in GC. This study provides insights into the mechanisms responsible for GC invasion and metastasis, and might lead to therapeutic targets for GC being identified in the near future.

However, more experiments should be performed to determine the mechanism by which GAL-1/*LGALS1* mediates TGF-β/Smad signaling activation. Taking the microenvironment of GC as the breakthrough point for the treatment of GC, the outcomes for patients with refractory GC could be improved.

## MATERIALS AND METHODS

### Patients

We enrolled 127 patients with primary gastric adenocarcinoma into the present study, of whom 89 were male and 38 were female, and their average age was 64.26 ± 9.98(range: 38–87) years. None of the patients had received neoadjuvant radiotherapy or chemotherapy, and all patients underwent radical gastrectomy at the Department of Gastrointestinal Surgery, Taizhou Clinical Medical School of Nanjing Medical University (Taizhou People’s Hospital) of Jiangsu province.

### Tumor and samples

For IHC, GC tissues and their matching adjacent gastric mucosa tissues were formalin-fixed and paraffin-embedded. For molecular analysis, GC tissues and their matching adjacent mucosal tissues were collected from 15 patients, and stored in liquid nitrogen. The Clinical Research Ethics Committee of Taizhou People’s Hospital (TZRY-EC-12-068) approved this study. All patients were provided with details regarding the assessment procedure, and all patients provided written, informed consent.

### Cell lines and their culture

SGC-7901 and MGC-803 (human gastric adenocarcinoma cell lines) were obtained from the Type Culture Collection of the Chinese Academy of Sciences (Shanghai, China). The cells were cultured in Roswell Park Memorial Institute (RPMI) medium (Thermo Fisher Scientific, Waltham, MA, USA) containing 1% (V/V) penicillin and streptomycin (Gibco, Grand Island, NY, USA), and 10% (V/V) fetal bovine serum (FBS; Thermo Fisher Scientific). The cells were cultured in a humidified atmosphere with 5% (V/V) CO_2_ at 37° C [25]. The cells were passaged via trypsinization when they reached 80% confluence.

### Lentiviral transduction

Lentiviral transduction was caried out according to a previously published protocol [25]. Genechem Co. Ltd (Shanghai, China) constructed the lentiviral vectors to overexpress and silence LGALS1. The short hairpin RNA (shRNA) sequences and the lentiviral vector were designed in a previous report [16]. SGC-7901 or MGC-803 cells were seeded at 5 × 10^4^ cells per well in 6-well plates before lentiviral transduction. Cells were transduced with the lentiviral vectors and 10 μg/mL polybrene (Sigma-Aldrich, St. Louis, MO, USA) at a multiplicity of infection of 10. At 12 h after transfection, the medium was replaced. To select stably transduced cell lines Puromycin (2 μg/mL; Sigma-Aldrich) was added. After 48 h, medium containing puromycin at 0.5 μg/mL was used to culture the stably transduced cells. After 72 h, a fluorescence microscope (OLYMPUS-U-HGLGPS-IX73, Olympus, Tokyo, Japan) was used to assess the transduction efficiency, which was further confirmed using qRT-PCR and WB.

### Reagents and antibodies

ITD-1, a TGF-β signaling pathway inhibitor, was purchased from Macklin (1884570, Shanghai, China). Abcam provided the anti-GAL-1 antibody (ab138513, Cambridge, UK); Cell Signaling Technology provided the anti-SMA-α antibody (56856, Danvers, MA, USA); anti-E-cadherin (bs-10009R), anti-Vimentin (bs-0756R), anti-TGF-β1 (bs-0086R), and anti-p-Smad2/3(bs-8853R) antibodies were purchased from Bioss (Beijing, China); Santa Cruz Biotechnology (Santa Cruz, CA, USA) provided the anti-glyceraldehyde phosphate dehydrogenase (GAPDH) antibody (sc-47724), the goat anti-rabbit IgG (sc-2357), the horseradish peroxidase (HRP)-conjugated goat anti-mouse IgG (sc-516102); Sigma Biotechnology (St. Louis, MO, USA) provided the MTT assay kit and dimethyl sulfoxide (DMSO).

### RNA extraction and qRT-PCR

Total RNA was extracted using an RNeasy Mini Kit (Invitrogen, Waltham, MA, USA), and cDNA was synthesized from RNA using a Reverse transcription kit (Takara, Shiga, Japan). The qPCR reactions were carried out using a SYBR Green dye kit (Roche Diagnostics, Mannheim, Germany) and the reaction products were analyzed using an iQ5 Multicolor Real-Time PCR Detection System (Bio-Rad, Hercules, CA, USA). The following thermocycling conditions were used: 95° C for 30 s; 40 cycles of 95° C for 5 s, 60° C for 30 s, and 72° C for 30 s. The reference control gene was GAPDH. The following primers were used: *LGALS1* (forward): GCTGAACCTGGGCAAAGACAG an GTGGCGGTTGGGGAACTT (producing a 247 bp amplicon); and *GAPDH* (forward) TGACTTCAACAGCGACACCCA) and (reverse) CACCCTGTTGCTGTAGCCAAA (producing a 121 bp amplicon).

### Western blotting

An extraction kit (Beyotime, Shanghai, China) was used to prepare total cell and nuclear extracts. 10% sodium dodecyl sulfate-polyacrylamide gel electrophoresis (SDS-PAGE) was used to separate 20 μg of the extracts, followed by transfer of the separated proteins to a nitrocellulose membrane (GE Healthcare Life Sciences, Pittsburgh, PA, USA). The proteins on the blots were probed with antibodies against GAL-1, E-cadherin, Vimentin, TGF-β1, p-Smad2/3, and GAPDH(dilution = 1:2000). Goat anti-rabbit immunoglobulin conjugated with HRP comprised the secondary antibody (dilution= 1:2000). A West Pico chemiluminescent Substrate (Pierce, Carlsbad, CA, USA) was used to visualize the immunoreactive protein bands, which were quantified using densitometric image analysis software (Image Master VDS; Pharmacia Biotech, Little Chalfont, UK). As an internal reference, GAPDH level were determined. All experiments were carried out three times independently.

### Histological examination and IHC evaluation

IHC was used to detect proteins in murine lung tissues, human GC tissues, and matching adjacent non-GC tissues according to our previous reports [14, 25]. Primary antibodies recognizing SMA-α (1:200), GAL-1 (1:200), E-cadherin (1:150), vimentin (1:150), TGF-β1 (1:150), and p-Smad2/3 (1:150) were incubated with the slides. Subsequent steps and the staining scores of the proteins of interest (GAL-1, SMA-α, p-Smad2/3, and TGF-β1) were the same as in our previous report [25]. Vimentin and E-cadherin staining was assessed as positive or negative according to the evaluation of the staining results as defined in our previous report [25].

### Wound-healing assay

GC cells were cultured to a confluent monolayer (80–90 %) in a 6-well plate, and then a wound was scored across the cell surface using a sterile plastic tip. Phosphate-buffered saline (PBS) was used to wash the plates three times to remove cellular debris. The plates were incubated with serum-free medium containing mitomycin C at 10 μg/mL to block proliferation. At 0 and 48 h, the wound was photographed. The assays were performed three times independently.

### Cell viability assay

The MTT assay was used to assess cell viability. Cells (5 × 10^3^ cells/well) were seeded into the well of a 96-well flat bottom plate and cultured overnight at 37° C in a 5% CO_2_ humidified atmosphere. The cells were then treated with ITD1 at various concentrations (5, 10, 15, and 20 μM). After 48 h, MTT was added at 20 μL per well and incubated for 4 h. Thereafter, the supernatant was removed, and the formazan crystals were dissolved using 150 μL of DMSO. A microplate reader (Model 550; Bio-Rad) was used to measure the absorbance at 490 nm.

### Cell invasion and migration assays

GC cell invasive and migratory abilities were assessed using 24-well Transwell units with polycarbonate filters (pore size, 8.0 μm; Corning, Corning, NY, USA). First, the upper Transwell inserts were coated with 100 μl of Matrigel basement membrane (BD Biosciences, San Diego, CA, USA) or left uncoated, and then added with 100 μL of serum-free RPMI medium. Then, 1× 10 ^5^ cells were seeded on the upper Transwell inserts. Medium (600 μL) containing 10% FBS as a chemoattractant was added to the lower chamber. For 24 h at 37° C, the cells were allowed to migrate or invade from the upper chamber, after which the non-migratory or non-invasive cells were removed. 4% paraformaldehyde was used to fix the filters, and a 0.05% crystal violet solution was used to stain the cells. In each sample, six fields were selected randomly to count the cells under a microscope (magnification = 100×). All assays were conducted three times independently.

### Animal models

The Comparative Medicine Centre of Yangzhou University (Yang Zhou, JiangSu, China) provided 5-week-old male athymic mic, which were used to construct the lung metastasis model and subcutaneous GC implantation model of GC. The Ethics Committee of Yang Zhou University approved the animal experiments (YZU-EC-JS2352). The mice were bred under pathogen-free conditions in a laminar flow cabinet. LGALS1-overexpressing (OE-LGALS1) MGC-803 cells, OE-LGALS1 MGC-803 treated with ITD1, and MGC-803 cells (wild-type control) were inoculated separately into the right flank or the tail vein of the athymic mice at 2 × 10^6^ cells per mouse (n = 6). In the subcutaneous model group, the mice were sacrificed on day 21, whereas in the lung metastasis model group, the mice were sacrificed on day 50. We harvested the subcutaneous GC tumors or lung for hematoxylin and eosin (H&E) staining, WB, and immunohistochemical staining.

### Statistical considerations

The statistical analyses were conducted using SPSS 20.0 (IBM Corp, Armonk, NY, USA). Means ± standard error were used to express the continuous variables. Based on the normal distribution of the data, comparisons between groups were performed using one-way analysis of variance (ANOVA) and Dunnett’s t test. To determine the relationship between two variables, Spearman’s or Pearson correlation coefficients were used. Residual analysis was used to determine whether the actual data were consistent with regression model assumptions or not. Values of *P* < 0.05 were regarded as statistically significant.

### Ethics approval and consent to participate

This clinical study was approved by the Clinical Research Ethics Committee of Taizhou People’s Hospital (TZRY-EC-12-068). All patients consented to participate in our study. The animal experiments were approved by the Ethics Committee of Yang Zhou University (YZU-EC-JS2352).

## Supplementary Material

Supplementary Figure 1
