# Supplementary material for: Fibroblastic galectin-1-fostered invasion and metastasis are mediated by TGF-β1-induced epithelial-mesenchymal transition in gastric cancer
Source: Aging (Albany NY). 2021 Jul 14;13(14):18464–81. doi: 10.18632/aging.203295 (PMC8351703; doi:10.18632/aging.203295)
Supplement: Supplementary Figure 1 [file aging-13-203295-s001.pdf]

SUPPLEMENTARY FIGURE

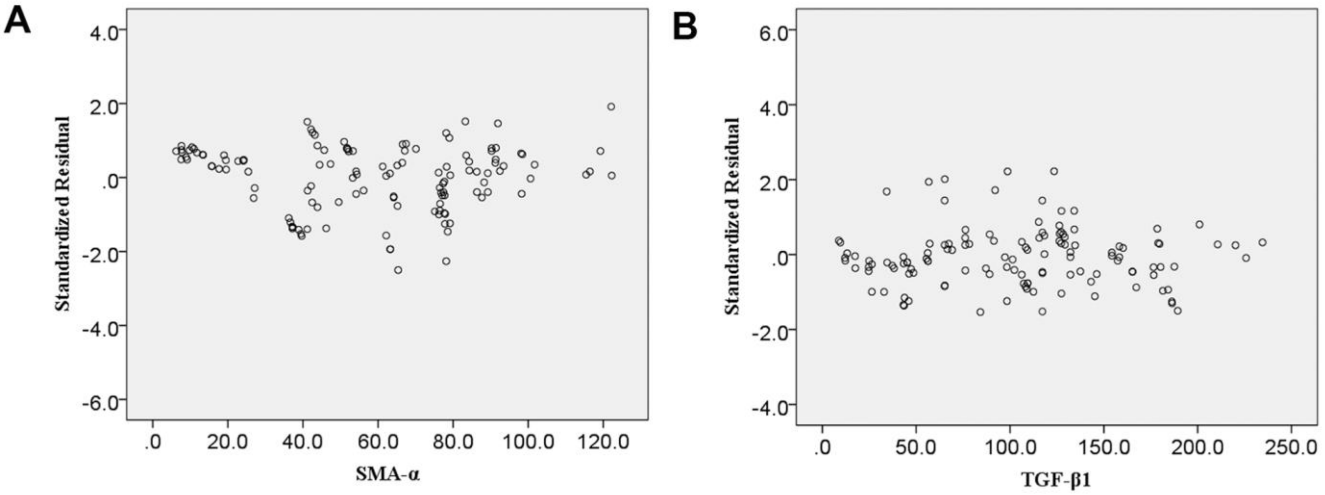

Supplementary Figure 1. Residual analysis on IHC signal proteins showing that the IHC signal proteins in this study fitted the regression model hypothesis. (**A**) Residual analysis showed that GAL-1/SMA-α proteins fitted the regression model hypothesis. (**B**) Residual analysis showing that GAL-1/TGF-β1 proteins fitted the regression model hypothesis.
